# Degree of adherence to plant-based diet and total and cause-specific mortality: prospective cohort study in the Million Veteran Program

**DOI:** 10.1017/S1368980022000659

**Published:** 2022-03-21

**Authors:** Dong D Wang, Yanping Li, Xuan-Mai T Nguyen, Rebecca J Song, Yuk-Lam Ho, Frank B Hu, Walter C Willett, Peter Wilson, Kelly Cho, J Michael Gaziano, Luc Djoussé

**Affiliations:** 1 Massachusetts Veterans Epidemiology Research and Information Center (MAVERIC), VA Boston Healthcare System, Boston, MA, USA; 2 The Channing Division of Network Medicine, Department of Medicine, Brigham and Women’s Hospital and Harvard Medical School, Boston, MA 02115, USA; 3 Departments of Nutrition, Harvard T. H. Chan School of Public Health, Boston, MA, USA; 4 Department of Medicine, Division of Aging, Brigham and Women’s Hospital, Boston, MA, USA; 5 Harvard Medical School, Boston, MA, USA; 6 Department of Epidemiology, Boston University School of Public Health, Boston, MA, USA; 7 Department of Epidemiology, Harvard T. H. Chan School of Public Health, Boston, MA, USA; 8 Atlanta VA Medical Center, Atlanta, GA, USA; 9 Emory Clinical Cardiovascular Research Institute, Atlanta, GA, USA

**Keywords:** Plant-based diet, Mortality, Public health

## Abstract

**Objective:**

To examine the association between adherence to plant-based diets and mortality.

**Design:**

Prospective study. We calculated a plant-based diet index (PDI) by assigning positive scores to plant foods and reverse scores to animal foods. We also created a healthful PDI (hPDI) and an unhealthful PDI (uPDI) by further separating the healthy plant foods from less-healthy plant foods.

**Setting:**

The VA Million Veteran Program.

**Participants:**

315 919 men and women aged 19–104 years who completed a FFQ at the baseline.

**Results:**

We documented 31 136 deaths during the follow-up. A higher PDI was significantly associated with lower total mortality (hazard ratio (HR) comparing extreme deciles = 0·75, 95 % CI: 0·71, 0·79, *P*_trend_ < 0·001]. We observed an inverse association between hPDI and total mortality (HR comparing extreme deciles = 0·64, 95 % CI: 0·61, 0·68, *P*_trend_ < 0·001), whereas uPDI was positively associated with total mortality (HR comparing extreme deciles = 1·41, 95 % CI: 1·33, 1·49, *P*_trend_ < 0·001). Similar significant associations of PDI, hPDI and uPDI were also observed for CVD and cancer mortality. The associations between the PDI and total mortality were consistent among African and European American participants, and participants free from CVD and cancer and those who were diagnosed with major chronic disease at baseline.

**Conclusions:**

A greater adherence to a plant-based diet was associated with substantially lower total mortality in this large population of veterans. These findings support recommending plant-rich dietary patterns for the prevention of major chronic diseases.

## What is already known on this topic?

Previous studies support the benefits of consuming plant-based diets for preventing cardiometabolic diseases such as coronary heart disease and type 2 diabetes and intermediate outcomes including obesity and adiposity-associated plasma biomarkers. However, studies on plant-based diets and mortality have been inconclusive and limited, especially for findings on cause-specific mortality due to cardiovascular disease and cancer.

## What this study adds

We found an inverse association between adherence to plant-based diets and mortality in a large cohort of US veterans. The association was stronger for the plant-based diet that emphasised healthy plant foods, and the risk of mortality was significantly elevated in participants with greater adherence to a plant-based diet that emphasied less-healthy plant foods. Our findings supported current dietary guidelines to increase intake of healthy plant foods at the expense of less-healthy plant foods and certain animal foods.

Plant-based diets are defined as a low frequency of consumption of animal foods and higher consumption of plant foods^(1)^. Previous studies support the benefits of consuming plant-based diets for preventing cardiometabolic disease end points such as coronary heart disease (CHD) and type 2 diabetes and intermediate outcomes including obesity and adiposity-associated plasma biomarkers^(2–8)^. Various authorities, including the American Heart Association (AHA)^(9)^ and the 2015 Dietary Guidelines for Americans^(10)^, recommend diets rich in plant foods to prevent major chronic diseases. To address the role of plant-based diets in overall health, analysis of total and cause-specific mortality as outcomes would be informative. However, studies on plant-based diets and mortality have been inconclusive and limited, especially for findings on cause-specific mortality due to cardiovascular disease (CVD) and cancer^(6,8,11–13)^.

Earlier studies defined plant-based diets dichotomously as ‘vegetarian’ *v*. ‘non-vegetarian’ by completely excluding certain groups of (e.g. red meat and poultry) or all animal foods^(7,8)^. However, it is challenging to study the health effects of vegetarian diets in US populations given the very low prevalence of vegetarianism (approximately 3 %)^(14)^. Alternatively, several recent studies employed *a priori* defined dietary indices to examine gradations of adherence to a plant-based diet^(2,3,15)^. The advantage of the indices is their broader applicability in US populations because recommendations of moderate, incremental dietary changes towards vegetarianism may be easier to adopt than more extreme, complete exclusion of animal foods. In addition, earlier investigations of vegetarian diets did not distinguish plant foods with divergent health effects, despite the fact that certain plant foods, such as refined grains^(16)^, potatoes^(17–19)^ and sugar-sweetened beverages^(20,21)^, were associated with an elevated risk of CVD, diabetes and mortality. To overcome this limitation, Satija et al. developed three plant-based diet indices (PDI), an overall PDI, a healthful PDI (hPDI) and an unhealthful PDI (uPDI), to assess the adherence to plant-based diets with consideration of the quality of plant foods and linked the indices to the risk of type 2 diabetes and CHD in health professional populations^(2,3,6)^. However, the earlier studies were conducted in populations that consist of older and predominantly White participants, mostly women and relatively high socio-economic status^(2–6)^. To address the limitations of the previous studies, we conducted this study in the VA Million Veteran Program (MVP), a newly launched prospective cohort study that consists primarily of male participants with a wide age range (19–104 years) and diverse socio-economic and racial/ethnic backgrounds. We hypothesise that PDI, hPDI and uPDI are divergently associated with total and cause-specific mortality in more than 0·3 million MVP participants with detailed dietary information.

## Methods

### Study population

MVP is a nationally representative, prospective cohort study of veterans designed to study genetic and non-genetic determinants of chronic diseases. MVP combines data from self-reported surveys, electronic health records and biospecimens. Details of the study design of MVP can be found elsewhere^(22)^. The enrolment of MVP participants began in 2011. As of 2020, 790 116 veterans were enrolled, and 351 892 participants have completed the baseline diet and lifestyle survey. Information on age, sex, race, education, body weight and height, alcohol consumption, physical activity and smoking status was collected through a self-reported survey at baseline. Health conditions, co-morbidities and medication use were obtained through Veterans Health Administration (VHA) electronic health records. We defined the baseline of this study as the time a participant completed the first lifestyle survey and the end of follow-up as the end of December 2018. All participants signed informed consent, and the Veterans Affairs Central Institutional Review Board approved the study protocol. We first excluded participants who did not provide dietary information, reported implausible dietary data (total energy intake < 1674 or > 16 736 kJ/d) or had more than 30 blanks on semi-quantitative food frequency questionnaire (SFFQ) at baseline. After this exclusion, a total of 327 480 participants were included. We then excluded 11 561 participants with relatively short follow-up, that is, those who responded to the lifestyle questionnaire after 2018. After the exclusions, the study population consisted of 315 919 participants (see online supplementary material, Supplementary Fig. 1).

### Dietary assessment

Dietary information was collected at baseline with an extensively validated SFFQ of sixty-one food items^(23)^. We asked how often, on average, the participant had consumed a specified portion size of each food over the preceding year on the SFFQ, with nine prespecified responses: ‘never or less than once a month’, ‘1–3 per month’, ‘once a week’, ‘2–4 per week’, ‘5–6’ per week, ‘once a day’, ‘2–3 per day’, ‘4–5 per day’ and ‘6+ per day’. We converted frequencies and portions of each food item to the average daily intake for each participant. We then calculated the average daily total energy intake by multiplying the frequency of consumption of each item by its energy content from the Harvard University Food Composition Database^(24)^ and summing across all foods. The SFFQ demonstrated reasonably well validity in assessing intakes of individual foods in our previous validation studies in the Health Professionals Follow-Up Study (HPFS) and the Nurses’ Health Study (NHS)^(25,26)^. For example, the average Pearson correlation coefficients corrected for within-person weekly variation comparing SFFQ-measured intakes to those measured by multiple 7-d food records (7DDR) ranged from 0·45 for nuts to 0·85 for tea/coffee in the HPFS^(25)^. In addition, we recently validated SFFQ-measured PDI against the indices measured by 7DDR; the correlations were 0·63 for PDI, 0·78 for hPDI and 0·73 for uPDI in the NHS, and 0·65 for PDI, 0·74 for hPDI and 0·77 for uPDI in the HPFS^(27)^.

### Definitions of plant-based diet indices

We applied the overall PDI, hPDI and uPDI to quantify each participant’s gradations of adherence to plant-based diets. Details of these indices can be found in our previous publications^(3)^. Briefly, we created sixteen food groups based on nutrient and culinary similarities of individual foods (see online supplementary material, Supplemental Table 1). The categorisation of healthy and less-healthy plant foods was based on the most recent empirical evidence of their associations with cardiometabolic disease (type 2 diabetes and CVD), certain cancers and intermediate conditions (obesity, hypertension, lipids and inflammation)^(10,17–21)^. All food groups within the same categories were given equal weight regardless of the strength of the evidence or the association of the individual foods with chronic disease risk. Healthy plant food groups included whole grains, fruits, vegetables, nuts, legumes and tea/coffee. Less-healthy plant food groups included fruit juices, sugar-sweetened beverages, refined grains, potatoes and sweets/desserts. Animal food groups included butter and lard, dairy, eggs, fish/seafood and meat (poultry and red meat). Third, we calculated quintiles of intake for each of the sixteen food groups and assigned component scores for each food group. For PDI, participants received 5 to 1 for their intake levels from the highest to the lowest quintiles of each plant food group (positive scoring). For animal foods, we reversed the scoring. For hPDI, we applied positive scoring to healthy plant food groups and reverse scoring to less-healthy plant food groups and animal food groups. For uPDI, positive scoring was applied to less-healthy plant food groups, and reverse scoring was applied to healthy plant food groups and animal food groups. Finally, we summed up component scores across the sixteen food groups to obtain the indices with the theoretical range of each index ranging from 16 to 80.

### Outcome ascertainment

The primary outcome of this study was total mortality. Deaths were identified through the National Death Index^(28)^. We defined three cause-specific mortality outcomes: deaths due to CVD, cancer (non-metastatic skin cancers excluded) and other causes. CVD mortality was defined based on the International Classification of Diseases, Tenth Revision, Clinical Modification (ICD-10-CM) codes I00 to I99. Cancer mortality was defined based on ICD-10-CM codes C00-C97.

### Statistical analysis

We categorised PDI, hPDI and uPDI into deciles based on their population distributions. Person-years of follow-up were calculated from baseline to the earliest time of death, loss to follow-up or the end of follow-up (which was December 2018). Cox proportional hazards models were applied to estimate hazard ratios (HR) and their 95 % CI of mortality, comparing participants in each category to the lowest category of a dietary index with simultaneous adjustment for covariables. For cause-specific mortality, we performed competing risk analysis using Cox proportional hazards models with a data augmentation method^(29)^. Multivariable model was adjusted for age (years: < 60, 60–70, > 70) and sex (male or female), race/ethnicity (non-Hispanic European American, African American or other), education level (≤ high school or GED, some colleague or college or above), income level (< $30 000, $30 000-$59 000 or ≥ $60 000) and marriage status (currently married or not), smoking status(current, former or never smoking), frequency of alcohol consumption (never, < 1 times/week or ≥ 1 times/week), frequency of exercise vigorously (never/rarely, 1–4 times/month, 2–4 times/week or ≥ 5 times/week), total energy intake (in quintiles) and BMI (<23·0, 23·0–24·9, 25·0–29·9, 30·0–34·9 or ≥35·0 kg/m^2^). In a separate model, we additionally adjusted for histories of hypertension, hypercholesterolemia, diabetes, cancer and CVD at baseline.

We used the median within each decile as a score variable and included it as a continuous variable in the model to quantify a linear trend; the Wald test was used for calculating *P*-values for linear trend. We also modelled the dietary indices continuously and calculated HR associated with every 10-unit increment in PDI, hPDI and uPDI. For example, an increase in nut and legume intake from 0 to 1 serving/d or a reduction in red and processed meat consumption from 1·5 servings to little consumption per d will result in a 10-unit improvement in the indices. To quantify non-linear dose–response relationship, restricted cubic splines with three knots were applied to flexibly model the association between the dietary indices and risk of mortality with the first percentile of each dietary score as the reference level^(30)^. We tested non-linearity in the dose–response relationship of the dietary indices with mortality by comparing the model with the linear term to the model with the linear and cubic spline terms using the likelihood ratio test.

We repeated the analyses on the associations of PDI scores with total mortality in European and African American groups separately and among participants who were free from and with histories of major chronic diseases, including diabetes, CVD and cancer at baseline. In addition, we conducted secondary analyses to test the robustness of our findings by examining the associations of PDI, hPDI and uPDI with total mortality in subgroups defined by smoking status, baseline age, baseline BMI and baseline histories of CVD, cancer, hypercholesterolemia, diabetes and hypertension. In another sensitivity analysis, we quantified associations of dietary scores with total mortality after excluding deaths within the first year of follow-up and participants with less than 1-year follow-up. The data analyses were performed using SAS software version 9.4 (SAS Institute, North Carolina) at a two-tailed α value of 0·05.

### Patient and public involvement

This research was done without patient involvement. Patients and the public were not invited to comment on the study design and were not consulted to develop patient-relevant outcomes or interpret the results. Patients and the public were not invited to contribute to the writing or editing of this document for readability or accuracy. The study did not receive funds to train or involve members of the community in the study design or interpretation of the results.

## Results

### Population characteristics

During a mean follow-up of 4 years, we documented 31 136 deaths, including 9751 deaths due to CVD, 9510 deaths due to cancer and 11 875 deaths due to other causes. At baseline, the study population had a mean age of 65·5 years (age range: 19 to 104 years) and consisted of 91·9 % men and 9·9 % African Americans (Table 1). Compared to the participants with a lower PDI, participants with a higher PDI were older, more physically active, and less likely to smoke, drink alcohol and have diabetes and hypertension. They also had higher education and income levels and a lower BMI at baseline.

**Table 1:** Baseline characteristics of 315 919 participants in the Million Veteran Program across deciles of plant-based diet indices

	Deciles of plant-based diet indices					
	PDI		hPDI		uPDI	
	Decile 1	Decile 10	Decile 1	Decile 10	Decile 1	Decile 10
*n*	34510	27498	34736	29651	30746	27976
PDI	37·6	59·3	45·7	51·6	48·1	47·5
uPDI	48·7	47·6	52·4	43·7	35·4	61·6
hPDI	44·8	50·9	36·3	59·6	51·7	43·9
Age, years	64·7	66·9	64·4	66·0	65·9	64·0
Men, %	91·7	92·5	94·2	88·2	89·6	91·7
European American, %	83·0	86·1	83·7	85·7	88·6	82·6
Married, %	55·6	64·6	58·5	60·1	64·7	54·2
Education level, %						
≤High school or GED	27·4	20·2	28·1	18·5	14·7	33·3
Some colleague	31·4	28·4	31·6	27·3	27·9	30·3
College or above	41·3	51·4	40·3	54·2	57·4	36·4
Annual family income, %						
<$30 000	37·1	32·9	37·9	31·9	28·1	41·1
$30 000–$59 000	35·1	36·3	36·3	34·3	34·5	35·7
≥$60 000	27·9	30·9	25·8	33·8	37·4	23·2
Smoking status, %						
Current smoking	6·1	5·3	7·3	4·3	5·1	7·0
Former smoker	65·7	63·2	64·3	63·9	64·7	62·8
Never smoking	28·2	31·5	28·4	31·8	30·2	30·2
Frequency of exercise vigorously, %						
Never/rarely	38·3	23·3	38·3	22·7	20·7	43·6
1–4 times/month	25·5	24·4	26·8	21·8	23·1	25·3
2–4 times/week	24·6	34·1	23·8	34·9	36·9	21·0
≥ 5 times/week	11·7	18·2	11·1	20·6	19·3	10·2
Frequency of alcohol drinking, %						
Never	42·0	44·5	44·9	44·4	36·9	51·3
<1 times/week	25·2	26·5	27·2	25·5	27·0	25·8
≥1 times/week	32·8	28·9	27·9	30·2	36·1	22·9
BMI, kg/m^2^	29·6	27·5	28·8	28·0	29·2	28·2
Diabetes, %	28·2	17·2	19·8	24·8	29·9	17·5
Hypertension, %	58·0	52·5	53·6	53·7	56·6	52·6
Hypercholesterolemia, %	50·2	50·4	47·2	52·0	51·6	47·5
Cancer, %	24·7	28·9	25·7	27·0	28·3	23·9
Cardiovascular disease, %	23·9	23·6	23·2	22·8	22·1	23·6
Food intake, servings/d						
Whole grains	0·2	0·7	0·2	0·5	0·7	0·1
Fruit	0·6	2·1	0·9	2·0	2·2	0·5
Vegetables	0·8	2·2	0·9	2·0	2·6	0·4
Nuts	0·2	0·7	0·3	0·6	0·7	0·2
Legume	0·2	0·8	0·3	0·7	0·9	0·2
Tea or coffee	1·2	2·3	1·2	2·2	2·5	0·9
Fruit juice	0·1	0·5	0·4	0·1	0·2	0·3
Sugar-sweetened beverages	0·2	0·6	0·9	0·1	0·1	0·8
Refined grains	0·5	1·3	1·4	0·5	0·7	1·0
Potatoes	0·3	0·8	0·9	0·3	0·4	0·6
Sweets and cake	0·3	1·3	1·3	0·4	0·6	1·0
Butter	0·5	0·2	0·7	0·1	0·7	0·2
Dairy	1·7	1·6	2·2	1·1	2·3	1·0
Eggs	0·6	0·3	0·6	0·3	0·8	0·2
Fish	0·2	0·2	0·2	0·2	0·3	0·1
All meat	1·7	1·7	2·4	1·0	2·2	1·2
Chicken	0·5	0·5	0·6	0·4	0·8	0·3
Red meat	0·7	0·7	1·1	0·4	0·9	0·5
Processed meat	0·5	0·4	0·7	0·2	0·5	0·3

PDI, plant-based diet index; hPDI, healthful PDI; UPDI, unhealthful PDI.

Unless otherwise indicated, data are expressed as means.

### Total mortality

**Fig. 1:**
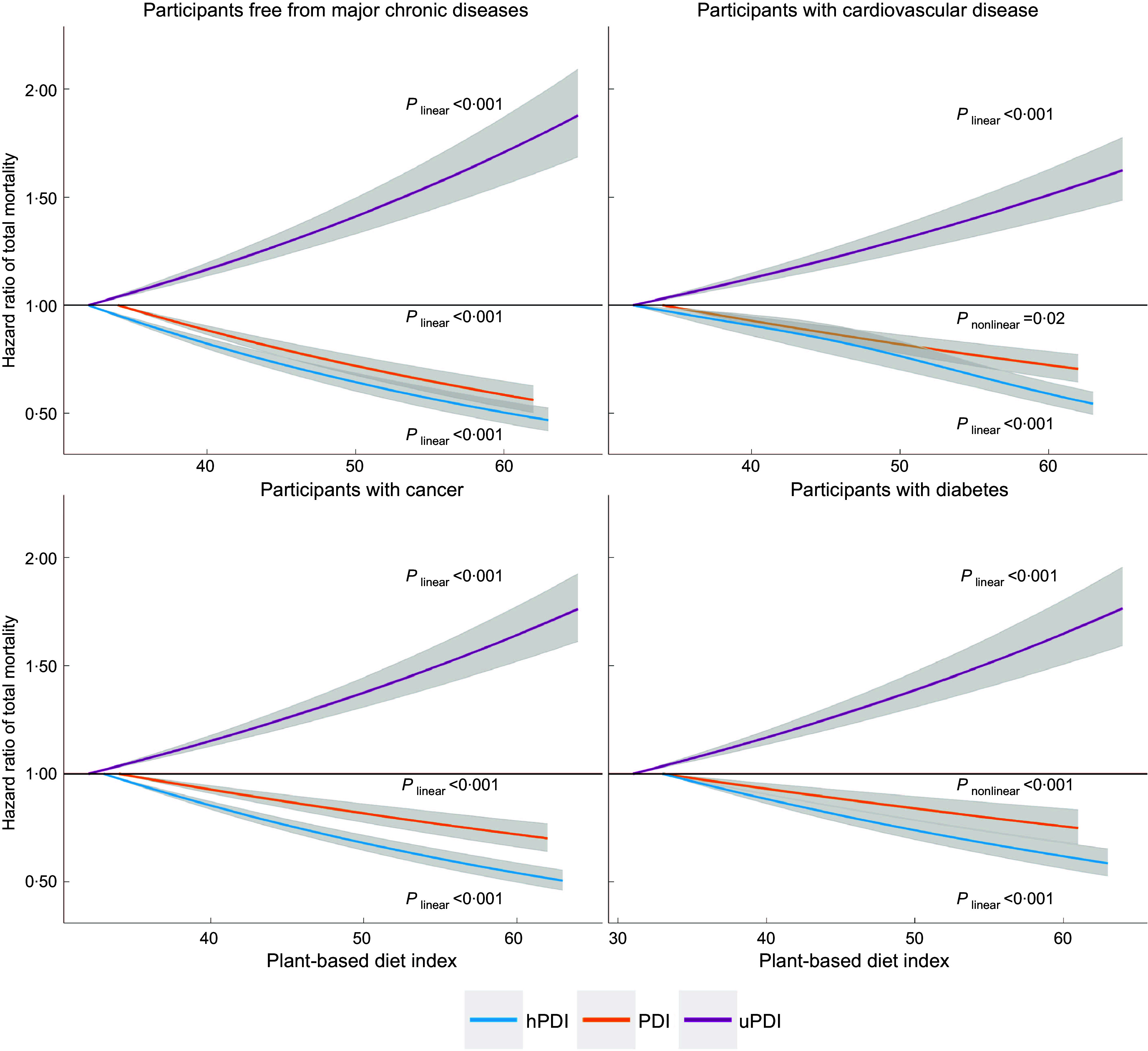
Dose–response relationship of plant-based diet indices with total mortality among participants free from and among those with a history of diabetes, CVD or cancer at baseline. The dose–response relationship was quantified by Cox proportional hazards models with restricted cubic spline with three knots specified. The first percentile of each dietary score was used as reference level for calculating hazard ratios. We tested non-linearity in the dose–response relationship by comparing the model with only the linear term to the model with the linear and the cubic spline terms and using the likelihood ratio test. All the models simultaneously adjusted for age (years: <60, 60–70, >70) and sex (male or female), race/ethnicity (non-Hispanic European American, African American or other), education level (≤ high school or GED, some colleague, or college or above), income level (< $30 000, $30 000–$59 000 or ≥ $60 000) and marriage status (currently married or not), smoking status(current, former or never smoking), frequency of alcohol consumption (never, < 1 times/week or ≥ 1 times/week), frequency of exercise vigorously (never/rarely, 1–4 times/month, 2–4 times/week or ≥ 5 times/week), total energy intake (in quintiles), BMI (< 23·0, 23·0–24·9, 25·0–29·9, 30·0–34·9 or ≥ 35·0 kg/m^2^), histories of hypertension, hypercholesterolemia, diabetes, cancer and CVD at baseline (yes *v*. no) (except among the same patients). The sample sizes were 148 244, 73 799, 74 213 and 85 149 for the analyses in participants free from major chronic diseases, with a history of diabetes, CVD or cancer at baseline, respectively. PDI, plant-based diet index; hPDI, healthful PDI; UPDI, unhealthful PDI

**Fig. 2:**
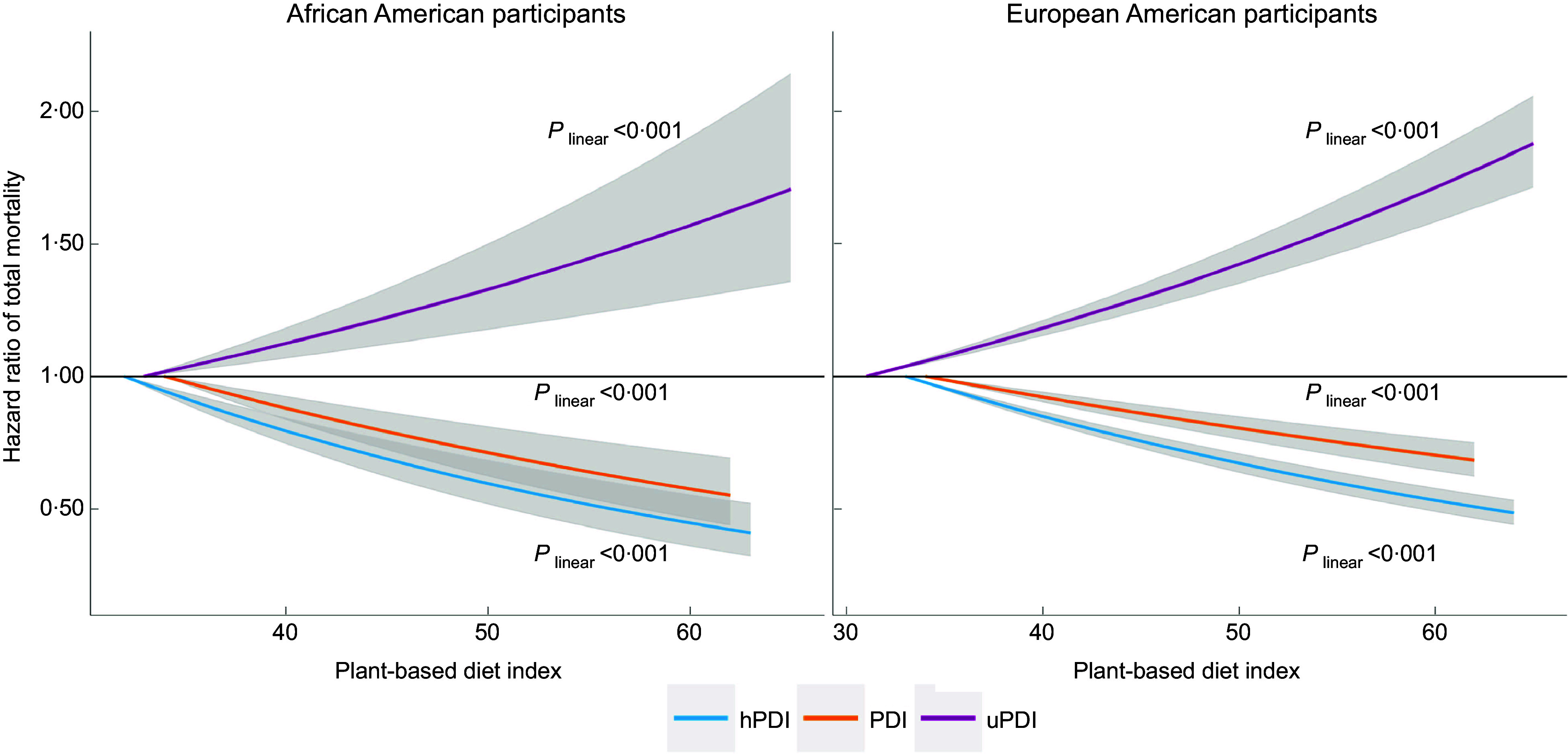
Dose–response relationship of plant-based diet indices with total mortality in African and European American participants. The dose–response relationship was quantified by Cox proportional hazards models with restricted cubic spline with three knots specified. The first percentile of each dietary score was used as reference level for calculating hazard ratios. We tested nonlinearity in the dose–response relationship of the dietary indices with mortality by comparing the model with only the linear term to the model with the linear and the cubic spline terms and using the likelihood ratio test. All the models simultaneously adjusted for age (years: < 60, 60–70, > 70) and sex (male or female), race/ethnicity (non-Hispanic European American, African American or other), education level (≤ high school or GED, some colleague or college or above), income level (< $30 000, $30 000–$59 000 or ≥ $60 000) and marriage status (currently married or not), smoking status (current, former or never smoking), frequency of alcohol consumption (never, < 1 times/week or ≥ 1 times/week), frequency of exercise vigorously (never/rarely, 1–4 times/month, 2–4 times/week or ≥ 5 times/week), total energy intake (in quintiles), BMI (< 23·0, 23·0–24·9, 25·0–29·9, 30·0–34·9, or ≥ 35·0 kg/m^2^), histories of hypertension, hypercholesterolemia, diabetes, cancer and CVD at baseline (yes *v*. no) (except among the same patients). The sample sizes were 28 018 and 241 374 for the analyses in African and European American participants, respectively. PDI, plant-based diet index; hPDI, healthful PDI; UPDI, unhealthful PDI

PDI was inversely associated with total mortality after multivariable adjustment for known and suspected confounding variables and risk factors (HR comparing extreme deciles = 0·75, 95 % CI: 0·71, 0·79, *P*_trend_ < 0·001; Table 2). Every 10-unit increment in PDI was associated with a 13 % lower total mortality (HR = 0·87, 95 % CI: 0·85, 0·89). Compared to the association for PDI, the inverse association of hPDI with total mortality was stronger; the highest decile of hPDI was associated with an HR of 0·64 (95 % CI: 0·61, 0·68, *P*_trend_ < 0·001) compared to the lowest decile. For uPDI, we observed a significant positive association with total mortality (HR comparing extreme deciles = 1·41, 95 % CI: 1·33, 1·49, *P*_trend_ < 0·001). When modelled continuously, every 10-unit increment in hPDI was associated with an HR of 0·81 (95 % CI: 0·80, 0·83), whereas every 10-unit increment in uPDI was associated with an HR of 1·15 (95 % CI: 1·13, 1·17). We observed slightly stronger associations for PDI, hPDI and uPDI in a subpopulation free from diabetes, CVD and cancer at baseline; the HR (95 % CI) of total mortality associated with a 10-unit increment in the indices were 0·82 (0·79, 0·86) for PDI, 0·79 (0·77, 0·83) for hPDI and 1·15 (1·11, 1·19) for uPDI (see online supplementary material, Supplemental Table 2). We found similar but slightly attenuated associations of PDI, hPDI and uPDI with total mortality in participants with baseline histories of diabetes, CVD and cancer (Fig. 1). The associations between the dietary indices and total mortality were not materially different between European American and Africa American participants (Fig. 2): *P* for interaction between the dietary indices and racial groups (European American or Africa American) was 0·03 for PDI, 0·39 for hPDI and 0·79 for uPDI. In addition, the associations between the dietary indices and total morality in Hispanic participants were comparable to those in the overall study population (see online supplementary material, Supplemental Fig. 2). The observed associations were generally consistent across subgroups defined by sex, age, family income, smoking status, alcohol consumption, physical activity level, weight status, and baseline histories of hypertension and hypercholesterolemia (see online supplementary material, Supplemental Fig. 2). In a sensitivity analysis that excluded deaths occurring during the first year of follow-up and participants who were followed for less than 1 year, the associations of PDI, hPDI and uPDI with mortality risks were similar to those in the primary analyses (see online supplementary material, Supplemental Table 3).

**Table 2:** Association of plant-based diet indices with total mortality in 315 919 participants from the Million Veteran Program

	Deciles of plant-based diet indices																					
	D1	D2		D3		D4		D5		D6		D7		D8		D9		D10		*P*_trend_	HR per 10-unit increment	
		HR	95 % CI	HR	95 % CI	HR	95 % CI	HR	95 % CI	HR	95 % CI	HR	95 % CI	HR	95 % CI	HR	95 % CI	HR	95 % CI		HR	95 % CI
Plant-based diet index																						
Median	38	42		44		46		48		49		50		52		55		59				
Deaths	3500	2374		3053		3634		3945		1874		3597		3048		3342		2769				
PY	124 765	88 673		118 051		142 841		154 256		77 511		147 355		125 608		137 259		115 365				
Model 1	Ref.	0·94	0·90, 0·99	0·88	0·84, 0·93	0·85	0·81, 0·89	0·85	0·81, 0·89	0·79	0·75, 0·84	0·78	0·75, 0·82	0·77	0·73, 0·80	0·75	0·72, 0·79	0·71	0·67, 0·74	<0·0001	0·84	0·83, 0·86
Model 2	Ref.	0·96	0·91, 1·01	0·90	0·86, 0·95	0·88	0·84, 0·92	0·88	0·84, 0·92	0·82	0·78, 0·87	0·81	0·77, 0·85	0·79	0·76, 0·84	0·78	0·74, 0·82	0·72	0·68, 0·76	<0·0001	0·85	0·84, 0·87
Model 3	Ref.	0·96	0·91, 1·01	0·91	0·87, 0·96	0·89	0·85, 0·94	0·89	0·85, 0·93	0·84	0·79, 0·89	0·83	0·79, 0·87	0·82	0·78, 0·86	0·80	0·76, 0·84	0·75	0·71, 0·79	<0·0001	0·87	0·85, 0·89
Healthful plant-based diet index																						
Median	37	41		44		46		47		48		50		52		55		59				
Cases	3904	3824		3254		3625		1896		3690		3299		2661		2689		2294				
PY	129 480	129 832		118 381		136 162		71 395		146 069		136 218		117 293		126 253		120 603				
Model 1	Ref.	0·93	0·89, 0·97	0·85	0·81, 0·89	0·82	0·78, 0·86	0·81	0·77, 0·86	0·76	0·73, 0·80	0·73	0·70, 0·77	0·69	0·66, 0·72	0·64	0·61, 0·67	0·57	0·54, 0·60	<0·0001	0·77	0·76, 0·79
Model 2	Ref.	0·98	0·94, 1·03	0·92	0·87, 0·96	0·90	0·86, 0·94	0·89	0·84, 0·94	0·84	0·81, 0·88	0·82	0·78, 0·86	0·78	0·74, 0·82	0·73	0·69, 0·77	0·65	0·62, 0·69	<0·0001	0·82	0·81, 0·84
Model 3	Ref.	0·98	0·94, 1·03	0·91	0·87, 0·96	0·89	0·85, 0·93	0·88	0·83, 0·93	0·83	0·80, 0·87	0·81	0·77, 0·85	0·77	0·73, 0·81	0·72	0·68, 0·76	0·64	0·61, 0·68	<0·0001	0·81	0·80, 0·83
Unhealthful plant-based diet index																						
Median	36	40		43		46		48		49		51		53		56		61				
Cases	2440	2533		3677		2933		3132		3240		3006		2801		4211		3163				
PY	118 512	113 242		154 425		119 798		125 746		122 922		114 878		103 248		150 641		108 271				
Model 1	Ref.	1·07	1·01, 1·13	1·14	1·08, 1·19	1·17	1·11, 1·23	1·20	1·14, 1·27	1·27	1·21, 1·34	1·29	1·23, 1·37	1·36	1·29, 1·44	1·42	1·36, 1·50	1·56	1·48, 1·64	<0·0001	1·20	1·18, 1·22
Model 2	Ref.	1·06	1·00, 1·12	1·11	1·06, 1·17	1·14	1·08, 1·20	1·14	1·08, 1·21	1·20	1·13, 1·26	1·20	1·14, 1·27	1·24	1·17, 1·31	1·27	1·21, 1·34	1·32	1·25, 1·39	<0·0001	1·12	1·10, 1·14
Model 3	Ref.	1·07	1·01, 1·13	1·14	1·08, 1·20	1·17	1·11, 1·23	1·19	1·13, 1·25	1·24	1·18, 1·31	1·25	1·18, 1·32	1·30	1·23, 1·37	1·34	1·28, 1·42	1·41	1·33, 1·49	<0·0001	1·15	1·13, 1·17

HR, hazard ratio; PY, person-year.

Model 1 adjusted for age (years: < 60, 60–70, > 70) and sex (male or female).

Model 2 further adjusted for race/ethnicity (non-Hispanic European American, African American or other), education level (≤ high school or GED, some colleague, or college or above), income level (< $30 000, $30 000–$59 000 or ≥ $60 000) and marriage status (currently married or not), smoking status (current, former or never smoking), frequency of alcohol consumption (never, < 1 times/week or ≥ 1 times/week), frequency of exercise vigorously (never/rarely, 1–4 times/month, 2–4 times/week or ≥ 5 times/week), total energy intake (in quintiles) and BMI (< 23·0, 23·0–24·9, 25·0–29·9, 30·0–34·9 or ≥ 35·0 kg/m^2^).

Model 3 further adjusted for histories of diabetes, hypertension, hypercholesterolemia, cancer and CVD at baseline (yes *v*. no).

### Cause-specific mortality

The PDI was inversely associated with cancer mortality (HR per 10-unit increment = 0·85, 95 % CI: 0·82, 0·88, *P*_trend_ < 0·0001) and CVD mortality (HR per 10-unit increment = 0·88, 95 % CI: 0·85, 0·91, *P*_trend_ < 0·0001, Table 3). A higher hPDI was significantly associated with lower mortality due to CVD (HR comparing extreme deciles = 0·69, 95 % CI: 0·63, 0·76, *P*_trend_ < 0·001) and cancer (HR comparing extreme deciles = 0·67, 95 % CI: 0·61, 0·74, *P*_trend_ < 0·001). We found significant positive associations between uPDI and both CVD and cancer mortality. Across extreme deciles, the uPDI was associated with CVD mortality (HR = 1·41 (95 % CI: 1·28, 1·55, *P*_trend_ < 0·001) and cancer mortality (HR = 1·36 (95 % CI: 1·24, 1·50, *P*_trend_ < 0·001). In a sensitivity analysis, we examined the associations of PDI with the risk of death due to specific cancer types (see online supplementary material, Supplemental Table 4). The associations with mortality due to four major cancer types, including digestive tract cancers, liver cancer, lung cancer and prostate cancer (in men only), were generally similar to that with mortality due to total cancer. In addition, we observed inverse associations of PDI and hPDI and positive association of uPDI with cancer mortality among cancer patients and CVD mortality among CVD patients, similar to the findings among participants without major chronic disease at baseline (see online supplementary material, Supplemental Table 5). In another secondary analysis, we found the associations of the dietary indices with the risk of death due to causes other than cancer and CVD were generally similar to those for total mortality (see online supplementary material, Supplemental Table 4).

**Table 3:** Association of plant-based diet indices with cause-specific mortality in 315 919 participants from the Million Veteran Program

		Deciles of dietary indices																					
		D1	D2		D3		D4		D5		D6		D7		D8		D9		D10			HR per 10-unit increment	
			HR	95 % CI	HR	95 % CI	HR	95 % CI	HR	95 % CI	HR	95 % CI	HR	95 % CI	HR	95 % CI	HR	95 % CI	HR	95 % CI	*P*_trend_	HR	95 % CI
		CVD Mortality																					
PDI	Cases	1107	709		949		1093		1270		566		1144		947		1045		921				
		Ref.	0·90	0·82, 0·99	0·89	0·82, 0·97	0·84	0·77, 0·92	0·89	0·82, 0·97	0·79	0·71, 0·87	0·82	0·75, 0·89	0·79	0·72, 0·86	0·78	0·71, 0·84	0·77	0·70, 0·84	<0·0001	0·88	0·85, 0·91
hPDI	Cases	1176	1171		1029		1113		598		1164		1046		848		863		743				
		Ref.	1·00	0·92, 1·08	0·95	0·88, 1·04	0·91	0·83, 0·98	0·92	0·83, 1·01	0·87	0·80, 0·94	0·84	0·78, 0·92	0·81	0·74, 0·89	0·76	0·69, 0·83	0·69	0·63, 0·76	<0·0001	0·84	0·81, 0·87
uPDI	Cases	757	826		1114		933		969		1054		909		904		1314		971				
		Ref.	1·13	1·02, 1·24	1·11	1·02, 1·22	1·20	1·09, 1·32	1·19	1·08, 1·31	1·30	1·19, 1·43	1·22	1·11, 1·35	1·35	1·23, 1·49	1·36	1·24, 1·49	1·41	1·28, 1·55	<0·0001	1·14	1·13, 1·19
		Cancer mortality																					
PDI	Cases	1017	739		936		1175		1252		605		1085		925		972		804				
		Ref.	1·03	0·95, 1·13	0·96	0·88, 1·05	0·99	0·91, 1·08	0·97	0·89, 1·05	0·92	0·84, 1·02	0·85	0·78, 0·93	0·85	0·77, 0·93	0·79	0·73, 0·87	0·74	0·68, 0·82	<0·0001	0·85	0·82, 0·88
hPDI	Cases	1164	1156		957		1124		583		1141		1031		804		844		706				
		Ref.	1·00	0·92, 1·08	0·90	0·82, 0·98	0·93	0·85, 1·01	0·91	0·82, 1·00	0·87	0·80, 0·94	0·85	0·78, 0·92	0·78	0·71, 0·86	0·76	0·69, 0·83	0·67	0·61, 0·74	<0·0001	0·83	0·81, 0·86
uPDI	Cases	788	753		1129		898		914		960		947		874		1265		982				
		Ref.	0·99	0·89, 1·09	1·08	0·99, 1·19	1·11	1·01, 1·22	1·08	0·98, 1·18	1·14	1·04, 1·25	1·22	1·11, 1·34	1·26	1·14, 1·38	1·25	1·15, 1·37	1·36	1·24, 1·37	<0·0001	1·14	1·11, 1·19
		Mortality due to other causes																					
PDI	Cases	1376	926		1168		1366		1423		703		1368		1176		1325		1044				
		Ref.	0·95	0·87, 1·03	0·88	0·81, 0·95	0·85	0·78, 0·91	0·81	0·75, 0·87	0·79	0·72, 0·86	0·79	0·73, 0·85	0·79	0·73, 0·85	0·79	0·73, 0·85	0·70	0·65, 0·76	<0·0001	0·85	0·83, 0·88
hPDI	Cases	1564	1497		1268		1388		715		1385		1222		1009		982		845				
		Ref.	0·96	0·89, 1·03	0·88	0·82, 0·95	0·85	0·79, 0·91	0·82	0·75, 0·90	0·78	0·72, 0·84	0·74	0·69, 0·80	0·72	0·67, 0·79	0·65	0·60, 0·70	0·59	0·54, 0·64	<0·0001	0·78	0·75, 0·80
uPDI	Cases	895	954		1434		1102		1249		1226		1150		1023		1632		1210				
		Ref.	1·10	1·00, 1·20	1·21	1·11, 1·32	1·20	1·10, 1·31	1·29	1·19, 1·41	1·28	1·18, 1·40	1·31	1·20, 1·43	1·29	1·18, 1·42	1·43	1·31, 1·55	1·48	1·36, 1·62	<0·0001	1·16	1·13, 1·19

PDI, plant-based diet index; hPDI, healthful plant-based diet index; uPDI, unhealthful plant-based diet index.

Models adjusted for age (years: < 60, 60–70, > 70) and sex (male or female), race/ethnicity (non-Hispanic European American, African American or other), education level (≤ high school or GED, some colleague, or college or above), income level (< $30 000, $30 000–$59 000 or ≥ $60 000) and marriage status (currently married or not), smoking status(current, former or never smoking), frequency of alcohol consumption (never, < 1 times/week or ≥ 1 times/week), frequency of exercise vigorously (never/rarely, 1–4 times/month, 2–4 times/week or ≥ 5 times/week), total energy intake (in quintiles), BMI (< 23·0, 23·0–24·9, 25·0–29·9, 30·0–34·9 or ≥ 35·0 kg/m^2^) and histories of diabetes, hypertension, hypercholesterolemia, cancer and CVD at baseline (yes *v*. no).

## Discussion

Our study represents the largest investigation of plant-based diets and mortality in a multi-ethnic cohort with diverse socio-economic backgrounds. In this population of more than 0·3 million US veterans, greater adherence to a plant-based diet was associated with lower risk of total, cancer and CVD mortality. We found that the associations of plant-based diets with mortality depend on the quality of plant foods. A higher hPDI was associated with a lower mortality risk, whereas a higher uPDI was associated with a higher mortality rate. Our findings were robust and remain largely unchanged among participants free from CVD and cancer and those diagnosed with major chronic disease at baseline. In addition, our results were consistent across different sex, age and racial/ethnic groups, supporting the generalisability of recommending plant-based diets in the US population.

Previous data on plant-based diets and total mortality have been inconclusive and limited^(6,8,11–13)^. Consistent with our findings, a recent study in NHS and HPFS found that 10-unit increases in PDI and hPDI during 12 years were associated with 8 % (HR = 0·92, 95 % CI: 0·89, 0·95) and 10 % (HR = 0·90, 95 % CI: 0·88, 0·93) lower total mortality, respectively, whereas a 10-unit increase in uPDI during 12 years was associated a 9 % (HR = 1·09, 95 % CI: 1·06, 1·12) higher total mortality^(5)^. In the Adventist Health Study 2, vegetarians, compared to non-vegetarians, had a 12 % lower risk of all-cause death during a follow-up of 7 years^(11)^. In contrast, in the National Health and Nutrition Examination Survey (NHANES) III and the European Prospective Investigation into Cancer and Nutrition Study (EPIC), neither the PDI nor the comparison between vegetarian and non-vegetarian diets was associated with total mortality^(8,13)^. These inconsistencies may be partly due to different population characteristics and variability in the adherence to plant-based diets in different studies. In line with previous studies that employed various nutritional metrics, such as glycaemic index, glycaemic load, fibre content and food sources of carbohydrate, to distinguish the healthfulness of plant foods^(16,31,32)^, we found that greater adherence to plant-based diets with emphasis on healthy plant foods (hPDI) was associated with a substantially lower risk of total mortality. In contrast, significantly higher total mortality was observed in participants with a higher uPDI. These divergent associations between the two versions of PDI highlighted the importance of considering the quality of plant foods when consuming and recommending plant-based diets.

Our data support the hypothesis that adherence to plant-based diets, particularly a plant-based diet with emphasis on healthy plant foods, reduces the risk of cancer mortality. Although limited studies have examined plant-based diets with total cancer outcomes^(6)^, substantial and consistent evidence from epidemiologic studies suggested that a dietary pattern rich in plant foods or a vegetarian diet was protective against colorectal cancer^(33,34)^. In addition, findings on hallmarks of a healthy plant-based diet with cancer risk have been consistent across studies. For example, based on extensive literature review, an updated report from the World Cancer Research Fund/American Institute for Cancer Research (WCRF/AICR) concluded with high gradings, that is, either as ‘convincing’ or ‘probable’, for evidence that low intakes of red/processed meat and high intakes of whole grains, non-starchy vegetables and fruit, and foods high in dietary fibres decrease the risk for cancer^(35)^. Our finding of an association between a higher PDI and improved cancer survival was consistent with previous data on healthy dietary patterns and mortality among cancer patients^(36–38)^ and provided additional evidence supporting the promotion of healthy eating as a means to ameliorate the adverse effects of cancer and its treatment, as recommended by various organisations such as the American Cancer Society (ACS) and WCRF/AICR^(35,39)^. In addition, we found a significant positive association between plant-based diets rich in less-healthy plant foods (uPDI) and cancer mortality. Such a finding is biologically plausible because an unhealthy plant-based diet is typically high in glycaemic load and index and added sugar, and low in dietary fibre, unsaturated fats and antioxidants, potentially leading to systemic inflammation that predisposes to the development of cancer and promotes all stages of tumorigenesis^(40)^.

When plant foods known to be associated with health benefits were emphasised, hPDI was associated with a lower risk of CVD mortality. Conversely, when the intake of less-healthy plant foods was emphasised, the opposite association with CVD mortality was observed. Our findings were broadly consistent with previous studies on both incidence and mortality of CVD^(2,6)^. For example, Satija et al. found that every 10-unit increment in hPDI was associated with a 12 % lower risk of CHD (HR = 0·88, 95 % CI: 0·85, 0·91), whereas every 10-unit increment in uPDI was associated with a 10 % higher risk of CHD (HR = 1·10, 95 % CI: 1·06, 1·14). There are several mechanisms through which a healthful plant-based diet could lower CVD risk. Such a diet is usually rich in dietary fibre, polyphenols, unsaturated fatty acids, micronutrients such as Mg, and low in saturated fat, Na:K ratio and glycaemic index. Thus, adherence to a healthful plant-based diet could lead to a lower risk of CVD through improving glycaemic control^(32)^, modulating lipid profile^(41)^ and decreasing chronic inflammation^(42,43)^. To our knowledge, our study is the first to report of a protective association between a plant-based diet and mortality among CVD patients. Previously studies reported that greater adherence to a Mediterranean diet, a dietary pattern that contains a variety of plant foods, was associated with less recurrence of myocardial infarction and longer survival in individuals with high cardiovascular risk^(44–46)^. Furthermore, our findings in both baseline CVD-free participants and CVD patients (Fig. 1) highlight the important role of a healthy plant-based diet in the management of CVD and emphasise how healthy eating patterns may influence prognosis of CVD.

Our secondary analysis revealed that the associations of PDI with the non-CVD and non-cancer mortality were similar to the associations for total mortality. The associations for deaths due to diabetes and respiratory disease were consistent with previous findings on healthy dietary patterns and the incident disease outcomes^(3,47)^, whereas the results for deaths due to chronic liver disease and cirrhosis, and renal failure are novel and therefore require confirmation in further studies.

Our study has several limitations. First, reverse causation is a possible explanation for our findings because people with chronic disease and poor health might change their habitual diet. However, our results remain unchanged in a subpopulation free from known major chronic diseases at baseline. Also, participants with a severe illness might change their diets towards ones generally perceived to be healthier and had a higher risk of mortality, leading to an attenuation in the associations between healthy diets and mortality, which would not explain away our findings. Second, the follow-up of our study was relatively short, which may not sufficiently capture the induction period of diet–mortality association. However, our study employed SFFQ to assess long-term usual diet and focused on overall dietary patterns rather than individual foods and nutrients. Usual dietary intake, especially dietary patterns, tends to be stable during adulthood. Therefore, the PDI represent the adherence to plant-based dietary patterns formed in early adulthood beyond the diet patterns captured at the start of follow-up. In addition, our findings were consistent with other studies of PDI with the incident disease during decades-long follow-ups^(2,6,48)^ and barely changed in the sensitivity analysis that excluded deaths during the first year of follow-up. Third, because our study is observational in nature, we are unable to establish causality. Fourth, although we carefully adjusted for many potential confounders, residual confounding could not be ruled out. Fifth, measurement errors are inevitable in estimates of dietary intakes. However, our adjustment for energy intake reduced the impact of measurement errors and controlled for potential confounding due to energy intake^(49)^. Lastly, the majority of the study participants were male. However, we found similar associations between PDI and mortality in men *v*. women. Strengths of the present study include the large sample size and high rates of follow-up. In addition, we addressed generalisability, a key limitation of previous work on plant-based diet^(2–6)^, by studying a population with a large proportion of men, a wide age range and diverse socio-economic and racial/ethnic backgrounds and testing the consistency of findings across various subgroups.

## Conclusions

We found an inverse association of higher adherence to PDI with mortality in a large cohort of US veterans. This inverse association was stronger for a PDI that emphasised healthy plant foods, and the risk of mortality was significantly elevated in participants with greater adherence to a plant-based diet that emphasised less-healthy plant foods. Increasing intakes of healthy plant foods at the expense of less-healthy plant foods and certain animal foods in the diet can confer substantial health benefits and should be a key message in the current dietary guidelines. Our findings strengthen the guidelines by addressing their generalisability in a unique study population with a wide age range and diverse socio-economic and racial/ethnic backgrounds.

## Supplementary material

For supplementary material accompanying this paper visit https://doi.org/10.1017/S1368980022000659. click here to view supplementary material
