# Supplementary material for: Degree of adherence to plant-based diet and total and cause-specific mortality: prospective cohort study in the Million Veteran Program
Source: Public Health Nutr. 2022 Mar 21;26(2):381–92. doi: 10.1017/S1368980022000659 (PMC13076072; doi:10.1017/S1368980022000659)
Supplement: Supplementary file 1 [file S1368980022000659sup.zip › S1368980022000659sup001.docx]

Supplementary File for: “Degree of Adherence to Plant-Based Diet and Total and Cause-Specific Mortality: Prospective Cohort Study in the Million Veteran Program”

Contents:

Page 1: cover page

Page 2: Supplementary Figure 1. Participant Flow Chart

Page 3: Supplementary Table 1: Food items constituting the 16 food groups and scoring of plant-based diet indices (PDI)

Page 4: Supplementary Table 2: Associations of plant-based diet indices with total mortality in 148,244 participants free from diabetes, cancer and cardiovascular disease at baseline.

Page 5: Supplementary Table 3: Associations of plant-based diet indices with total mortality after excluding deaths within the first year of follow-up and participant with less than one-year follow-up.

Page 6: Supplementary Table 4: Associations of plant-based diet indices with cause-specific mortalities.

Page 7: Supplementary Table 5: Associations of plant-based diet indices (PDI) with cancer mortality among cancer patients and CVD mortality among CVD patients.

Page 8: Supplementary Figure 2. Association of plant-based diet index and total mortality in subgroups.

**Supplementary Figure 1. Participants flow chart**

790,116 veterans were enrolled VA Million Veteran Program as of 2020

351,892 participants had completed the baseline diet and lifestyle survey.

327,480 participants with plausible food frequency questionnaire

excluded 11,561 participants who responded

to the lifestyle questionnaire after 2018

the study population consisted of 315,919 participants

**Supplementary Table 1.** **Food items constituting the 16 food groups and** **scoring of plant-based diet indices (PDI)**

| **Food groups** | **Food items** | **Scoring for each quintile of PDIs**  **(Q1, Q2, Q3, Q4, Q5)** | | |
| --- | --- | --- | --- | --- |
|  |  | **PDI** | **hPDI** | **uPDI** |
| **Healthy plant food groups** |  |  |  |  |
| Whole grains | Dark bread | 1,2,3,4,5 | 1,2,3,4,5 | 5,4,3,2,1 |
| Fruits | Fresh apples or pears, oranges, peaches, apricots or plums bananas, and other fruits | 1,2,3,4,5 | 1,2,3,4,5 | 5,4,3,2,1 |
| Vegetables | Tomatoes or tomato juice, broccoli, cabbage, cauliflower, or brussels sprouts, carrots, corn, yams or sweet potatoes, spinach or collard greens, and yellow (winter) squash | 1,2,3,4,5 | 1,2,3,4,5 | 5,4,3,2,1 |
| Nuts | Nuts and peanut butter | 1,2,3,4,5 | 1,2,3,4,5 | 5,4,3,2,1 |
| Legumes | String beans, peas or lima beans, and beans or lentils | 1,2,3,4,5 | 1,2,3,4,5 | 5,4,3,2,1 |
| Tea/coffee | Coffee, tea | 1,2,3,4,5 | 1,2,3,4,5 | 5,4,3,2,1 |
| **Less healthy plant food groups** |  |  |  |  |
| Fruit juices | Orange juice or grapefruit juice | 1,2,3,4,5 | 5,4,3,2,1 | 1,2,3,4,5 |
| Sugar-sweetened beverages | Carbonated beverage with sugar, Hawaiian Punch, lemonade, or other non-carbonated fruit drink | 1,2,3,4,5 | 5,4,3,2,1 | 1,2,3,4,5 |
| Refined grains | White bread (slice), including pita bread, cold breakfast cereal, and rice or pasta, e.g., spaghetti, noodles, etc. | 1,2,3,4,5 | 5,4,3,2,1 | 1,2,3,4,5 |
| Potatoes | French fried potatoes, baked, boiled or mashed potatoes, potato chips | 1,2,3,4,5 | 5,4,3,2,1 | 1,2,3,4,5 |
| Sweets/desserts | Chocolate, candy, pie, cake, and cookies | 1,2,3,4,5 | 5,4,3,2,1 | 1,2,3,4,5 |
| **Animal food groups** |  |  |  |  |
| Butter | Butter (pat), added to food or bread | 5,4,3,2,1 | 5,4,3,2,1 | 5,4,3,2,1 |
| Dairy | Skim or low-fat milk, whole milk, yogurt, ice cream, cottage or ricotta cheese and other cheese | 5,4,3,2,1 | 5,4,3,2,1 | 5,4,3,2,1 |
| Eggs | Eggs | 5,4,3,2,1 | 5,4,3,2,1 | 5,4,3,2,1 |
| Fish/seafood | Fish | 5,4,3,2,1 | 5,4,3,2,1 | 5,4,3,2,1 |
| Meat | Chicken or turkey, bacon, hot dogs, processed meats, e.g., sausage, salami, bologna, etc., liver, hamburger, beef, pork, or lamb | 5,4,3,2,1 | 5,4,3,2,1 | 5,4,3,2,1 |
| **Range of scores** |  | 16~80 | 16~80 | 16~80 |

**Supplementary Table 2.** Associations of plant-based diet indices with total mortality in 148,244 participants free from diabetes, cancer and cardiovascular disease at baseline.

|  | Deciles of Dietary Indices | | | | | | | | | |  | HR per |
| --- | --- | --- | --- | --- | --- | --- | --- | --- | --- | --- | --- | --- |
|  | D1 | D2 | D3 | D4 | D5 | D6 | D7 | D8 | D9 | D10 | *P* _trend_ | 10-unit increment |
| **Plant-based diet Index** | | | | | | | | | | | | |
| Deaths | 1002 | 652 | 826 | 1008 | 1078 | 508 | 976 | 824 | 928 | 748 |  |  |
| PYs | 55851 | 40187 | 54123 | 65844 | 70914 | 36084 | 68146 | 59373 | 64368 | 55214 |  |  |
| HR (95%CI) | Ref. | 0.92  (0.83, 1.02) | 0.86  (0.78, 0.94) | 0.86  (0.78, 0.93) | 0.83  (0.76, 0.91) | 0.79  (0.71, 0.88) | 0.78  (0.71, 0.85) | 0.73  (0.67, 0.81) | 0.75  (0.69, 0.83) | 0.65  (0.59, 0.71) | <0.0001 | 0.82  (0.79, 0.86) |
| **Healthful Plant-based diet Index** | | | | | | | | | | | | |
| Cases | 1228 | 1119 | 889 | 1013 | 496 | 965 | 853 | 698 | 694 | 595 |  |  |
| n | 64632 | 62341 | 55514 | 63337 | 33066 | 66314 | 61057 | 53428 | 56282 | 54134 |  |  |
| HR (95%CI) | Ref. | 0.94  (0.86, 1.02) | 0.86  (0.79, 0.94) | 0.86  (0.79, 0.93) | 0.80  (0.72, 0.89) | 0.78  (0.71, 0.85) | 0.76  (0.70, 0.83) | 0.71  (0.65, 0.79) | 0.67  (0.61, 0.74) | 0.61  (0.55, 0.67) | <0.0001 | 0.79  (0.77,0.83) |
| **Unhealthful Plant-based diet Index** | | | | | | | | | | | | |
| Cases | 588 | 688 | 976 | 777 | 832 | 841 | 868 | 779 | 1212 | 989 |  |  |
| n | 49781 | 49837 | 68605 | 53987 | 57691 | 56969 | 53993 | 49381 | 73568 | 56291 |  |  |
| HR (95%CI) | Ref. | 1.12  (1.01, 1.25) | 1.14  (1.03, 1.27) | 1.18  (1.06, 1.31) | 1.17  (1.05, 1.30) | 1.21  (1.08, 1.34) | 1.31  (1.18, 1.46) | 1.31  (1.17, 1.46) | 1.35  (1.22, 1.5) | 1.46  (1.31, 1.63) | <0.0001 | 1.15 (1.11,1.19) |

HR (95%CI): Hazard Ratio (95% confident interval) adjusted for age (years: <60, 60-70, >70), sex (male or female), race/ethnicity (non-Hispanic white, African American or other), education level (≤ high school or GED, some colleague, or college or above), income level (< $30,000, $30,000-$59,000 or ≥ $60,000) and marriage status (currently married or not), smoking status(current, former or never smoking), frequency of alcohol consumption (never, < 1 times/week or ≥ 1 times/week), frequency of exercise vigorously (never/rarely, 1-4 times/month, 2-4 times/week, or ≥ 5 times/week), total energy intake (in quintiles), body mass index (<23.0, 23.0-24.9, 25.0-29.9, 30.0-34.9, or ≥35.0 kg/m^2^) and histories of hypertension, and hypercholesterolemia at baseline (yes vs. no).

**Supplementary Table 3.** Associations of plant-based diet indices with total mortality after excluding deaths within the first year of follow-up and participant with less than one-year follow-up.

|  | Deciles of Dietary Indices | | | | | | | | | |  | HR per |
| --- | --- | --- | --- | --- | --- | --- | --- | --- | --- | --- | --- | --- |
|  | D1 | D2 | D3 | D4 | D5 | D6 | D7 | D8 | D9 | D10 | *P* _trend_ | 10-unit increment |
| **Plant-based diet Index** | | | | | | | | | | | | |
| Deaths | 3060 | 2088 | 2681 | 3280 | 3553 | 1665 | 3214 | 2778 | 3038 | 2541 |  |  |
| PYs | 122484 | 87262 | 116176 | 140759 | 152104 | 76433 | 145455 | 124027 | 135681 | 114193 |  |  |
| HR (95%CI) | Ref. | 0.96  (0.91, 1.02) | 0.91  (0.86, 0.96) | 0.91  (0.87, 0.96) | 0.91  (0.86, 0.95) | 0.84  (0.79, 0.89) | 0.83  (079, 0.88) | 0.84  (0.79, 0.88) | 0.81  (0.77, 0.86) | 0.77  (0.72, 0.81) | <0.0001 | 0.88  (0.86, 0.90) |
| **Healthful Plant-based diet Index** | | | | | | | | | | | | |
| Cases | 3412 | 3386 | 2923 | 3241 | 1698 | 3316 | 2957 | 2393 | 2453 | 2119 |  |  |
| n | 127386 | 127805 | 116755 | 134206 | 70378 | 144080 | 134354 | 115783 | 124727 | 119101 |  |  |
| HR (95%CI) | Ref. | 0.99  (0.94, 1.04) | 0.93  (0.89, 0.98) | 0.91  (0.86, 0.95) | 0.89  (0.84, 0.95) | 0.85  (0.81, 0.89) | 0.82  (0.78, 0.86) | 0.79  (0.74, 0.83) | 0.74  (0.70, 0.78) | 0.67  (0.63, 0.71) | <0.0001 | 0.83  (0.81, 0.84) |
| **Unhealthful Plant-based diet Index** | | | | | | | | | | | | |
| Cases | 2220 | 2297 | 3335 | 2630 | 2819 | 2892 | 2671 | 2483 | 3742 | 2809 |  |  |
| n | 116738 | 111650 | 152247 | 118106 | 124034 | 121239 | 113307 | 101864 | 148609 | 106780 |  |  |
| HR (95%CI) | Ref. | 1.07  (1.01, 1.13) | 1.14  (1.08, 1.20) | 1.15  (1.09, 1.22) | 1.18  (1.11, 1.25) | 1.22  (1.15, 1.29) | 1.23  (1.16, 1.30) | 1.27  (1.20, 1.35) | 1.32  (1.25, 1.40) | 1.39  (1.31, 1.47) | <0.0001 | 1.14  (1.12, 1.16) |

HR (95%CI): Hazard Ratio (95% confident interval) adjusted for age (years: <60, 60-70, >70), sex (male or female), race/ethnicity (non-Hispanic white, African American or other), education level (≤ high school or GED, some colleague, or college or above), income level (< $30,000, $30,000-$59,000 or ≥ $60,000) and marriage status (currently married or not), smoking status(current, former or never smoking), frequency of alcohol consumption (never, < 1 times/week or ≥ 1 times/week), frequency of exercise vigorously (never/rarely, 1-4 times/month, 2-4 times/week, or ≥ 5 times/week), total energy intake (in quintiles), body mass index (<23.0, 23.0-24.9, 25.0-29.9, 30.0-34.9, or ≥35.0 kg/m^2^) and histories of hypertension, hypercholesterolemia, cancer, and cardiovascular disease at baseline (yes vs. no).

**Supplementary Table 4:** Associations of plant-based diet indices (PDI) with cause-specific mortalities (HR (95%CI), *P* value, per 10-unit increment)^*^

| **Causes of mortality** | **Cases** | **PDI** | **Healthy PDI** | **Unhealthy PDI** |
| --- | --- | --- | --- | --- |
| Digestive tract cancers | 1000 | 0.84 (0.75, 0.95), *P*=0.004 | 0.76 (0.68, 0.85), *P*<0.0001 | 1.16 (1.05, 1.28), *P*=0.003 |
| Live cancer | 575 | 0.84 (0.72, 0.97), *P*=0.02 | 0.75 (0.65, 0.87), *P*=0.0001 | 1.03 (0.90, 1.17), *P*=0.66 |
| Lung cancer | 2486 | 0.86 (0.80, 0.93), *P*<0.0001 | 0.85 (0.79, 0.91), *P*<0.0001 | 1.15 (1.08, 1.22), *P*<0.0001 |
| Prostate cancer (men only) | 1025 | 0.83 (0.74, 0.94), *P*=0.004 | 0.87 (0.78, 0.98), *P*=0.02 | 1.00 (0.90, 1.11), *P*=0.94 |
| All other and unspecified cancers | 4424 | 0.87 (0.82, 0.92), *P*<0.0001 | 0.81 (0.77, 0.85), *P*<0.0001 | 1.13 (1.08, 1.19), *P*<0.0001 |
| Heart diseases | 7535 | 0.89 (0.85, 0.92), *P*<0.0001 | 0.82 (0.79, 0.86), *P*<0.0001 | 1.15 (1.11, 1.19), *P*<0.0001 |
| Cerebrovascular diseases | 1140 | 0.93 (0.84, 1.04), *P*=0.22 | 0.86 (0.78, 0.95), *P*=0.004 | 1.17 (1.06, 1.28), *P*=0.001 |
| Other cardiovascular diseases | 1076 | 0.90 (0.80, 1.01), *P*=0.07 | 0.85 (0.76, 0.94), *P*=0.003 | 1.17 (1.07, 1.29), *P*=0.001 |
| Diabetes mellitus | 1031 | 0.78 (0.70, 0.88), *P*<0.0001 | 0.77 (0.59, 0.85), *P*<0.0001 | 1.14 (1.04, 1.26), *P*=0.006 |
| Chronic liver disease and cirrhosis | 388 | 0.73 (0.60, 0.87), *P*=0.0007 | 0.71 (0.59, 0.85), P=0.0002 | 1.38 (1.19, 1.62), *P*<0.0001 |
| Renal failure | 515 | 0.74 (0.62, 0.87), *P*=0.0002 | 0.70 (0.60, 0.81), *P*<0.0001 | 1.25 (1.09, 1.43), *P*=0.002 |
| Other chronic diseases | 3046 | 0.84 (0.79, 0.90), *P*<0.0001 | 0.77 (0.73, 0.82), *P*<0.0001 | 1.18 (1.11, 1.25), *P*<0.0001 |
| Respiratory diseases | 3458 | 0.84 (0.79, 0.90), *P*<0.0001 | 0.80 (0.76, 0.85), *P*<0.0001 | 1.13 (1.08, 1.20), *P*<0.0001 |
| All other causes | 3437 | 0.88 (0.83, 0.94), *P*<0.0001 | 0.85 (0.80, 0.90), *P*<0.0001 | 1.17 (1.11, 1.23), *P*<0.0001 |

^*^Hazard Ratio (95% confident interval) adjusted for age (years: <60, 60-70, >70), sex (male or female), race/ethnicity (non-Hispanic white, African American or other), education level (≤ high school or GED, some colleague, or college or above), income level (< $30,000, $30,000-$59,000 or ≥ $60,000) and marriage status (currently married or not), smoking status(current, former or never smoking), frequency of alcohol consumption (never, < 1 times/week or ≥ 1 times/week), frequency of exercise vigorously (never/rarely, 1-4 times/month, 2-4 times/week, or ≥ 5 times/week), total energy intake (in quintiles), body mass index (<23.0, 23.0-24.9, 25.0-29.9, 30.0-34.9, or ≥35.0 kg/m^2^) and histories of hypertension, hypercholesterolemia, cancer, and cardiovascular disease at baseline (yes vs. no).

**Supplementary Table 5:** Associations of plant-based diet indices (PDI) with cancer mortality among cancer patients and CVD mortality among CVD patients (HR (95%CI), *P* value, per 10-unit increment) ^*^

| **Causes of mortality** | **PDI** | **Healthy PDI** | **Unhealthy PDI** |
| --- | --- | --- | --- |
| Cancer mortality among cancer patients | 0.85 (0.81-0.89)  *P* _trend_<0.0001 | 0.79 (0.75-0.83) _trend_<0.0001 | 1.11 (1.06-1.16)  *P* _trend_<0.0001 |
| CVD mortality among CVD patients | 0.92 (0.88-0.97)  *P* _trend_<0.0001 | 0.85 (0.81-0.89)  *P* _trend_<0.0001 | 1.12 (1.08-1.17)  *P* _trend_<0.0001 |

^*^Hazard Ratio (95% confident interval) adjusted for age (years: <60, 60-70, >70), sex (male or female), race/ethnicity (non-Hispanic white, African American or other), education level (≤ high school or GED, some colleague, or college or above), income level (< $30,000, $30,000-$59,000 or ≥ $60,000) and marriage status (currently married or not), smoking status(current, former or never smoking), frequency of alcohol consumption (never, < 1 times/week or ≥ 1 times/week), frequency of exercise vigorously (never/rarely, 1-4 times/month, 2-4 times/week, or ≥ 5 times/week), total energy intake (in quintiles), body mass index (<23.0, 23.0-24.9, 25.0-29.9, 30.0-34.9, or ≥35.0 kg/m^2^) and histories of hypertension, hypercholesterolemia, cancer, and cardiovascular disease at baseline (yes vs. no).

**
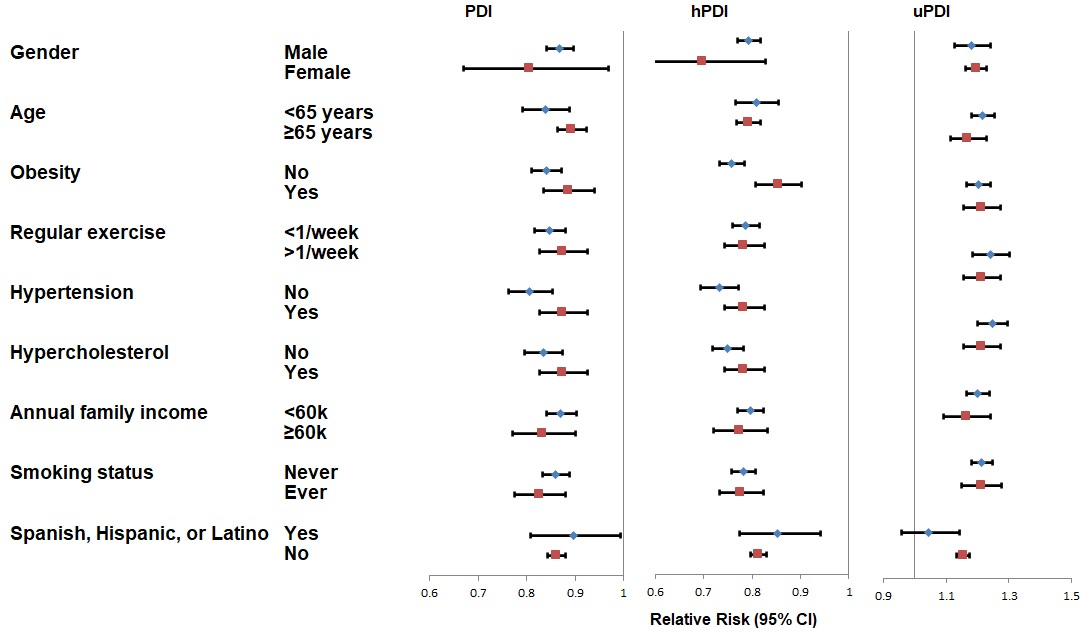
**

**Supplementary Figure 2.** Association of plant-based diet index and total mortality in subgroups.

Models adjusted for age (continuous) and sex (male or female), race/ethnicity (non-Hispanic European American, African American or other), education level (≤ high school or GED, some colleague, or college or above), income level (< $30,000, $30,000-$59,000 or ≥ $60,000) and marriage status (currently married or not), smoking status(current, former or never smoking), frequency of alcohol consumption (never, < 1 times/week or ≥ 1 times/week), frequency of exercise vigorously (never/rarely, 1-4 times/month, 2-4 times/week, or ≥ 5 times/week), total energy intake (in quintiles), body mass index (<23.0, 23.0-24.9, 25.0-29.9, 30.0-34.9, or ≥35.0 kg/m^2^) and histories of diabetes, hypertension, hypercholesterolemia, cancer, and cardiovascular disease at baseline (yes vs. no) except the variables of stratification.
